# The Anti-TNF-α Antibody Infliximab Inhibits the Expression of Fat-Transporter-Protein FAT/CD36 in a Selective Hepatic-Radiation Mouse Model

**DOI:** 10.3390/ijms16034682

**Published:** 2015-03-02

**Authors:** Gesa Martius, Silke Cameron, Margret Rave-Fränk, Clemens F. Hess, Hendrik A. Wolff, Ihtzaz A. Malik

**Affiliations:** 1 Department of Gastroenterology and Endocrinology, University Medical Center Goettingen, Robert-Koch-Strasse 40, Goettingen 37075, Niedersachsen, Germany; E-Mails: gesa.martius@med.uni-goettingen.de (G.M.); silke.cameron@med.uni-goettingen.de (S.C.); 2 Department of Radiotherapy and Radiooncology, University Medical Center Goettingen, Robert-Koch-Strasse 40, Goettingen 37075, Niedersachsen, Germany; E-Mails: mfraenk@med.uni-goettingen.de (M.R.-F.); cfhess@med.uni-goettingen.de (C.F.H.); hendrik.wolff@med.uni-goettingen.de (H.A.W.)

**Keywords:** irradiation, fat accumulation, liver, FAT/CD36, TNF-α, infliximab

## Abstract

Previously, we reported a radiation-induced inflammation triggering fat-accumulation through fatty-acid-translocase/cluster of differentiation protein 36 (FAT/CD36) in rat liver. Furthermore, inhibition of radiation-induced FAT/CD36-expression by anti-tumor necrosis factor-α (anti-TNF-α) (infliximab) was shown *in vitro*. The current study investigates fat-accumulation in a mouse-model of single-dose liver-irradiation (25-Gray) and the effect of anti-TNF-α-therapy on FAT/CD36 gene-expression. Mice livers were selectively irradiated *in vivo* in presence or absence of infliximab. Serum- and hepatic-triglycerides, mRNA, and protein were analyzed by colorimetric assays, RT-PCR, Immunofluorescence and Western-Blot, respectively. Sudan-staining was used demonstrating fat-accumulation in tissue. In mice livers, early (1–3 h) induction of TNF-α-expression, a pro-inflammatory cytokine, was observed. It was followed by elevated hepatic-triglyceride level (6–12 h), compared to sham-irradiated controls. In contrast, serum-triglyceride level was decreased at these time points. Similar to triglyceride level in mice livers, Sudan staining of liver cryosections showed a quick (6–12 h) increase of fat-droplets after irradiation. Furthermore, expression of fat-transporter-protein FAT/CD36 was increased at protein level caused by radiation or TNF-α. TNF-α-blockage by anti-TNF-α showed an early inhibition of radiation-induced FAT/CD36 expression in mice livers. Immunohistochemistry showed basolateral and cytoplasmic expression of FAT/CD36 in hepatocytes. Moreover, co-localization of FAT/CD36 was detected with α-smooth muscle actin (α-SMA^+^) cells and F4/80^+^ macrophages. In summary, hepatic-radiation triggers fat-accumulation in mice livers, involving acute-phase-processes. Accordingly, anti-TNF-α-therapy prevented early radiation-induced expression of FAT/CD36 *in vivo*.

## 1. Introduction

The liver is the key organ in the body energy homeostasis, due to its ability to metabolize and distribute fatty acids [1]. It is also able to synthesize fatty acids (*de novo* lipogenesis) as well as to degrade or convert incoming fatty acids from the circulating system [2]. The parenchymal cells of the liver, *i.e.*, hepatocytes, are mainly responsible for the fat metabolism [3].

The accumulation of lipids in the liver can be the result of increased lipolysis from adipose tissue, increased intake of dietary fat, *de novo* hepatic lipogenesis, and decreased free fatty acid oxidation and hepatic very low density lipoprotein-triglycerides secretion [4]. Pathologically, excessive intrahepatic triglyceride storage is termed steatosis, which may result from a variety of liver treatments and diseases including alcohol, medication and hepatitis C. The established histological criterion for steatosis is the presence of more than 5% of triglycerides stored in hepatocytes [5].

Fat homeostasis is controlled by a number of factors including different enzymes, transcription factors, membrane and/or intracellular proteins involved in transport, synthesis and degradation of fat [6,7,8,9].

The recently discovered fatty-acid-translocase/cluster of differentiation protein 36 (FAT/CD36) plays an active role in hepatic fat metabolism. Free fatty acids are taken up by hepatocytes via transport proteins like the transporter fatty acid translocase (FAT/CD36) [6,10]. FAT/CD36 is a membrane-bound glycoprotein present on platelets, mononuclear phagocytes, adipocytes, hepatocytes, and myocytes [6,11,12,13].

Two isoforms of FAT/CD36 have been described. The active FAT/CD36 protein isoform at 88 kDa has two transmembrane domains [14]. It has a variety of ligands, including fatty acids [15]. Furthermore, an inactive non-glycosylated isoform of FAT/CD36 protein can be detected at 54 kDa [15]. A ligand-specific aspect of FAT/CD36 signaling involves its capacity to deliver biologically active lipids to cells.

There is evidence that fatty acids are oxidized, and bind to FAT/CD36. However, the exact mechanism of fatty acid uptake into liver cells remains unclear [16,17].

Radiation-induced inflammation in healthy and diseased tissue is a now well established fact. Clinically, radiation-induced liver disease (RILD) is a serious clinical complication due mainly to vessel damage [18], and, as a consequence, secretion of inflammatory mediators. Moreover, ionizing radiation is recently reported to alter the expression of proteins involved in the fat metabolism pathway in parallel to accumulation of fat in adipose tissue of rats [19]. This could lead to non-alcoholic liver disease (NALD) and radiation-induced fibrosis (RIF) [20]. However, changes in hepatic triglyceride (TG) levels and related proteins after targeted liver radiation have not been fully investigated so far.

Corresponding to accumulation of fat, induction of pro-inflammatory cytokines is also well documented in hepatocellular damage or fibrosis models [21,22]. Among the cytokines, production of TNF-α is one of the earliest events in hepatic inflammation after liver irradiation [23].

As the liver is the pivotal metabolic organ, hepatic impairment may have serious consequences. In our previous work, we have shown that single-dose percutaneous irradiation of rat liver induces periportal inflammation [24] and changes the gene expression of proteins including those of iron metabolism [25] and inflammatory mediators [23,26]. Furthermore, we recently showed that a single-dose liver irradiation can trigger intracellular fat accumulation in the rat liver in parallel to induction of FAT/CD36 expression. This effect may be conveyed by radiation-induced TNF-α expression. We also showed that FAT/CD36 was induced by irradiation or TNF-α *in vitro* in a human cell line (human monocytic cell line U937). In contrast, anti-TNF-α treatment reduced this up-regulating effect of irradiation *in vitro*. In the current study, we extended our previous knowledge and showed that irradiation could trigger fat accumulation in the mouse liver, as observed in the rat model. Furthermore, we could show that anti-TNF-α treatment reduced the radiation-caused induction of FAT/CD36 in mouse liver *in vivo*.

## 2. Results

### 2.1. Real Time PCR Analysis of Tumor Necrosis Factor-α (TNF-α) in Irradiated Mice Liver

**Figure 1:**
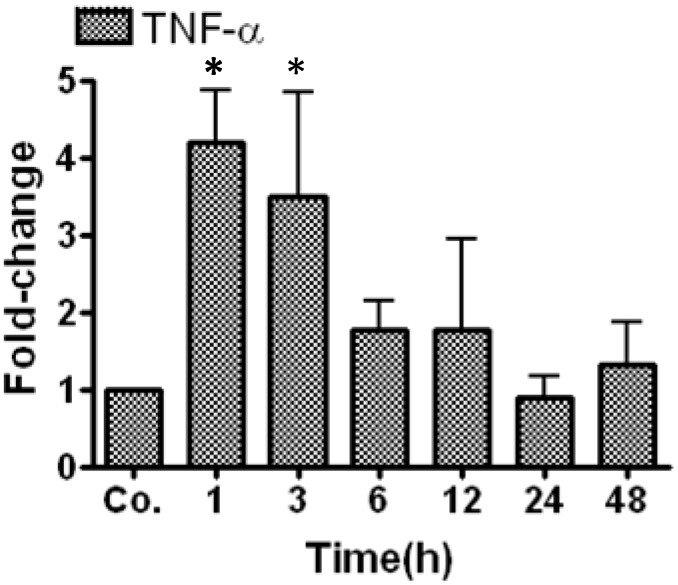
qRT-PCR analysis of total mRNA isolated from mice livers after irradiation. Data are shown as fold-changes in mRNA expression of *TNF-α* at the various time-points relative to sham-irradiated controls for each time-point. qRT-PCR was normalized by using two housekeeping genes: β-*actin* and glyceraldehyde-3-phosphate dehydrogenase (*GAPDH*). Results represent means ± standard error mean (S.E.M) of five experiments; * *p <* 0.05 *n =* 5. Co. = control.

The mRNA expression of TNF-α, a major pro-inflammatory cytokine, was analyzed in mouse liver tissue at 1, 3, 6, 12, 24 and 48 h after irradiation using real-time PCR analysis. A quick and maximum upregulation of TNF-α mRNA (4.2 ± 0.66-fold) was detected after 1 and 3 h. The gene expression of TNF-α then decreased but remained above the control levels until 12 h (Figure 1).

### 2.2. Changes in Triglycerides (TG) Level in Mice Livers and Serum after Irradiation

**Figure 2:**
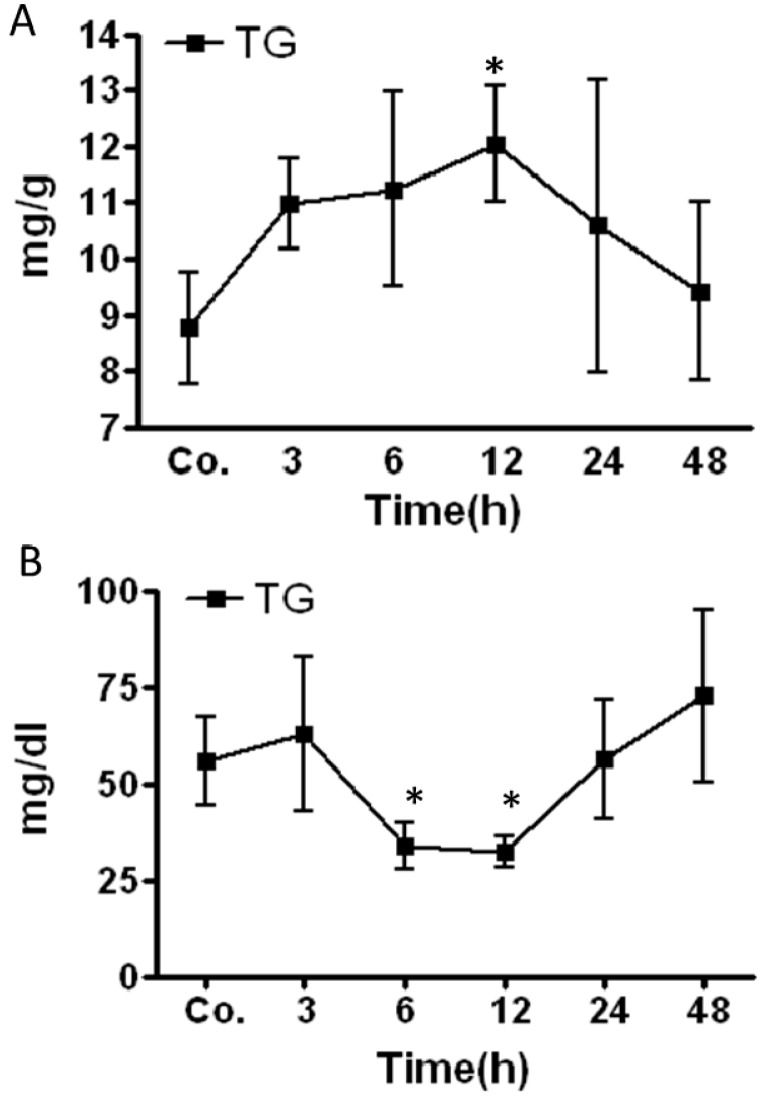
Changes in triglyceride (TG) concentrations in liver tissue lysate (**A**) and serum (**B**) of irradiated mice compared to the sham-irradiated controls for each time point. Results represent mean value ± S.E.M. of five animals * p < 0.05*.*

An increase in the TG level was detected after irradiation in mice livers. The elevated TG-content started to be prominent at 3 h with further increase at 6 h. The increase became significant at 12 h (12 ± 1 mg/g liver tissue) post-RT compared to sham-irradiated controls (8.7 mg/g liver tissue). After reaching that maximum, the TG concentration decreased (Figure 2A). In contrast, the TG concentration in mice serum decreased after irradiation compared to sham-irradiated controls. The TG concentration in the serum significantly decreased at 6 h (56.11 ± 11.5 mg/dL *vs.* control 34 ± 6 mg/dL) and reached a minimum at 12 h (32.2 ± 4.2 mg/dL) post-RT. After 12 h post RT the concentration of TG returned to normal values which were reached at 24 h post RT (Figure 2B). Furthermore, combination of irradiation and infliximab (IFX) showed a minor reduction in total TG levels caused by irradiation alone however it did not reach to significance (data not shown).

### 2.3. Early Accumulation of Fat in Irradiated Mice Liver Tissue

**Figure 3:**
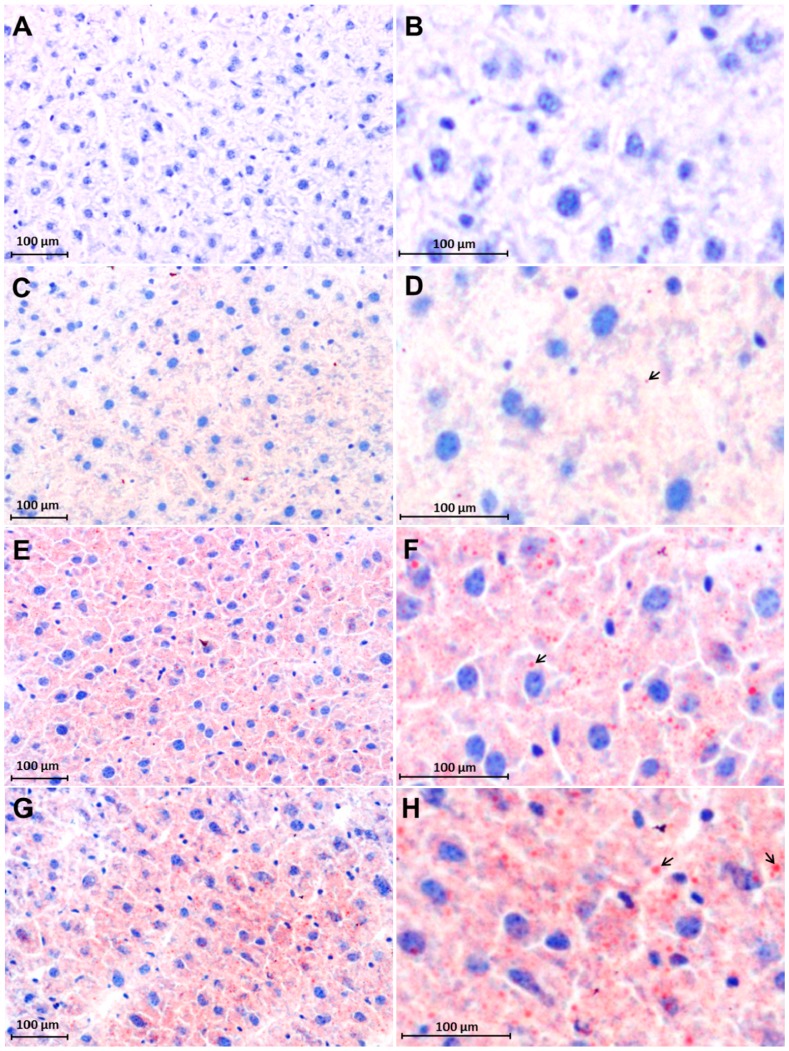
Accumulation of intracellular fat visualized with Sudan III staining in cryosections of livers of sham-irradiated control and irradiated mice. Negative control (**A**,**B**); Sham-irradiated controls (**C**,**D**); 6 h post-RT (**E**,**F**) and 12 h post-RT (**G**,**H**). The intracellular accumulation of fat droplets (red color) increased with the time after irradiation. (**B**,**D**,**F** and **H**) are magnified areas of (**A**,**C**,**E** and **G**); The arrows in pictures (**D**,**F** and **H**) show intracellular lipid droplets. Nuclei are stained blue. Results are representative photographs of three animals and six slides per time point (original magnification, ×200).

Sudan III staining showed an increase in fat droplets after RT in comparison to the corresponding sham-irradiated control mice liver cryosections. A very few fat droplets were noticed in Sham irradiated controls (Figure 3C,D) whereas number and size of fat droplets were increased after irradiation. Fat droplets were mainly stained red within hepatocytes. The increased fat droplets accumulation was clearly visible after 6 h post-RT (Figure 3E,F) with a maximum at 12 h (Figure 3G,H) compared to a sham-irradiated control (Figure 3C,D). Negative control with counterstaining of only hematoxylin showed no positivity for Sudan III (Figure 3A,B).

### 2.4. Changes in FAT/CD36 Protein Level in Mice Livers by Western Blot Analysis

**Figure 4:**
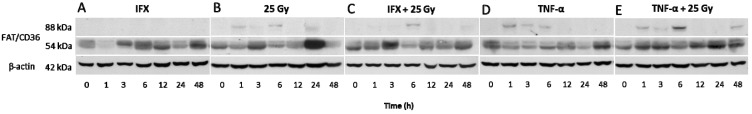
Western blot analysis of fatty-acid-translocase/cluster of differentiation protein 36 (FAT/CD36) from total protein extracts of mice livers. infliximab (IFX)-treated (**A**, IFX); irradiated (**B**, 25 Gray(Gy)); IFX-treated and irradiated (**C**, IFX + 25 Gy); TNF-α-treated (**D**, TNF-α); and TNF-α-treated and irradiated (**E**, TNF-α + 25 Gy) mice samples were analyzed by using anti-FAT/CD36 antibody at different time points. Two bands, ~88 (active isoform) and ~54 (inactive isoform), were detected. β*-actin* was used as loading control. Results are representative for three animals.

Western blot was performed to analyze the time-dependent change in protein content of FAT/CD36 in the group of single administration of IFX (G6), irradiated group (G2), irradiated and IFX-administered (G4), single administration of TNF-α (G5) and irradiated and TNF-α-administered (G3), respectively (Figure 4). As previously described [27], FAT/CD36 is built as a non-active form (54 kDa) and then post-translationally glycosylated to its active form of 88 kDa.

A variable increase in protein level of the non-glycosylated isoform of FAT/CD36 (54 kDa) was observed in comparison to untreated controls. The 88 kDa isoform was not detected in the livers of sham-irradiated and IFX-treated mice (Figure 4A). Post-RT, an early increase of the 88 kDa isoform (active isoform) at 1 and 6 h (Figure 4B) was detected. In contrast, the radiation effect was prevented early at 1–3 h when anti-TNF-α was administered prior to irradiation. At 6 h, an expression of the 88 kDa isoform was observed (Figure 4C). Similar to the observed increase after irradiation, TNF-α administration led to an increase of the active FAT/CD36 protein (Figure 4D) at 1 to 6 h. Furthermore, a synergetic effect of TNF-α and irradiation was observed at 1 to 6 h with a maximum at 6 h. (Figure 4E).

### 2.5. Localization of FAT/CD36 in the Liver Using Immunofluorescent Staining

**Figure 5:**
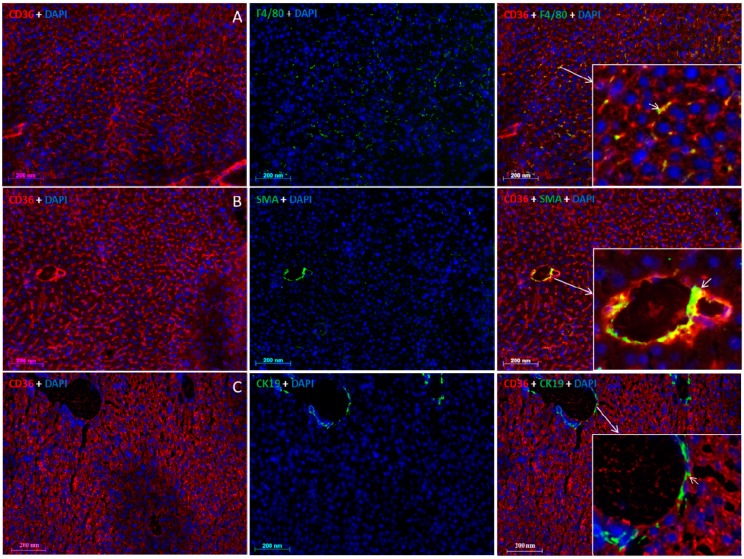
Immunofluorescence double-staining of mice liver cryosections. Row **A**: Monoclonal antibody against FAT/CD36 (**A** **left**, red), monoclonal antibody directed against F4/80 (**A** **middle**, green) and merged FAT/CD36 and F4/80 (**A** **right**); Row **B**: Monoclonal antibody against FAT/CD36 (**B** **left**, red), monoclonal antibody directed against SMA (**B** **middle**, green) and merged FAT/CD36 and SMA (**B** **right**); Row **C**: Monoclonal anti-FAT/CD36 antibody (**C** **left**, red), polyclonal antibody directed against CK19 (**C**, **middle**, green) and merged FAT/CD36 and CK19 (**C** **right**). Nuclei staining was done with (DAPI). The insets show higher magnification. Results show the representative picture of three animals and six slides (original magnification, ×200).

**Figure 6:**
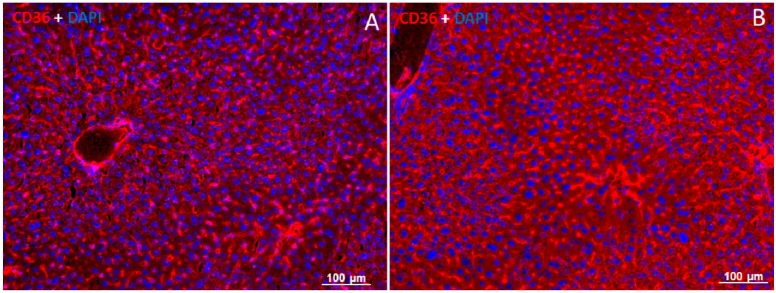
Immunofluorescence detection of FAT/CD36 in liver cryosections of sham-irradiated control and irradiated mice. Sham-irradiated controls (**A**); 6 h post-RT (**B**). Staining with the monoclonal anti-FAT/CD36 antibody showed an increased protein expression of FAT/CD36 (red) after irradiation. Counter staining of the nuclei was done with 4',6-diamidino-2-phenylindole (DAPI). Results show the representative picture of three animals and six slides (original magnification, ×100).

By means of immunofluorescence staining, using a mouse monoclonal antibody against FAT/CD36, a fluorescent signal for FAT/CD36 was detected in the basolateral membrane and the cytoplasm of hepatocytes (Figure 5). Positivity of FAT/CD36 was furthermore noticed in F4/80− and SMA-positive cells, corresponding to FAT/CD36-co-localization in macrophages (F4/80+) (Figure 5 row A) and vessel walls (SMA+) (Figure 5 row B). A very weak positivity of FAT/CD36 was also detected in bile duct cells (CK−19+) (Figure 5 row C). Similar to Western blot analysis, an increased expression of FAT/CD36 was detected after 6 h post-RT compared to sham irradiated controls (Figure 6A,B). The negative control showed no specific signals (not shown).

## 3. Discussion

Previously, we reported the induction of an inflammatory response in rat liver by single-dose liver irradiation [24]. A major pro-inflammatory cytokine induced by liver irradiation is TNF-α [26]. The increase in TNF-α expression is accompanied by increased fat accumulation [28]. Furthermore, we and others showed that fat accumulation in rat liver is reflected by increased expression of genes involved in fatty acid metabolism as well as increase in fatty acid translocase, a fat transport protein (FAT/CD36) [7,28,29].

Therefore, the aim of this work was to ascertain if selective percutaneous liver irradiation could increase fat transport into mice livers similar to what was observed in rat livers [28]. Furthermore, we investigated whether anti-TNF-α (infliximab, IFX) treatment could reverse the radiation-induced expression of fat transporter protein FAT/CD36 *in vivo*.

In our mouse model, we could show increased hepatic TG levels, whilst TG serum levels were decreased. The accumulation of fat was further confirmed by Sudan staining. Accordingly, increased protein levels of the fat transporter protein FAT/CD36 were observed after mouse liver irradiation as well as after TNF-α treatment in mice. This effect was further enhanced when TNF-α was injected before mice liver irradiation. In contrast, the induction of FAT/CD36 expression by irradiation was prevented at early time points by anti-TNF-α (infliximab) therapy in mice.

These results are similar to our previous study where we showed in a human monocyte cell-line (U937) *in vitro* that anti-TNF-α (infliximab), antibody against soluble TNF-α, inhibits FAT/CD36 protein expression, thus preventing the increase of FAT/CD36 caused by TNF-α. In agreement, several previous studies reported that anti-TNF-α therapy is helpful to inhibit the development of steatohepatitis in patients with severe alcoholic steatohepatitis and nonalcoholic fatty liver disease in rodents and humans [30,31].

Another important aspect of the present study was to show that FAT/CD36 was not only expressed in the hepatocytes but was also observed in liver macrophages (*i.e.*, Kupffer cells), smooth muscle cells and weak expression in the bile duct cells. The late increase of FAT/CD36 after irradiation combined with anti-TNF-α treatment might result from FAT/CD36 expression by these cells, *i.e.*, liver macrophages, and not by hepatocytes.

The liver regulates the metabolism of glucose, proteins and fat. As the liver is the main organ for metabolism, its functional impairment has serious consequences. An inequality among fatty acid uptake, synthesis, oxidative and secretory pathways of fatty acids leads to hepatic steatosis [4] or steatohepatitis, which in turn may induce liver fibrosis and as a consequence leads to cirrhosis. The complicated process of fatty acid uptake is controlled by several proteins. Among them, the role of FAT/CD36 is crucial.

Previous studies reported that overexpression of FAT/CD36 confers increased fatty acid and lipoprotein influx and/or utilization [32]. Accordingly, it is associated with hepatic steatosis or metabolic disorders [33]. Increased expression of FAT/CD36 has also been reported in experimental models of NAFLD and patients [34]. Interestingly, ablation of FAT/CD36-mediated lipid uptake into liver or muscle prevented lipotoxicity in several animal models [7,35]. These studies confirm the importance of FAT/CD36 in hepatic lipid uptake, similarly in human and animal models.

Clinically, radiation-induced liver disease (RILD) is a serious complication [36] mainly due to radiation-induced damage of microcirculation, redox-stress mechanisms and inflammatory responses. Although a relation between hepatic inflammation and fat accumulation has already been established [1], an interaction between hepatic fat accumulation (through up-regulation of transporter mechanisms) and the radiation-induced inflammatory response (mediated by cytokine secretion) has not been investigated so far. Irradiation-triggered tissue fat accumulation with disturbance of metabolic pathways has only been reported in extrahepatic (*i.e.*, mice gonadal adipose) tissue after irradiation [19]. It has further been suggested, that FAT/CD36 plays a role in long-chain fatty acid uptake not only into the cell, but also into the mitochondria and thus is directly related to the energy metabolism of the cell, its up-regulation inducing an increased capacity for fatty acid oxidation [37]. Mitochondrial fatty acid oxidation is the source of increased production of reactive oxygen species (ROS) [38], which could be true in our current study as increased ROS production after irradiation was observed in this model (Malik *et al.*, manuscript in preparation). Furthermore, a direct effect of radiation on mitochondria, resulting in mitochondrial ROS production has also been proposed [39]. Mitochondrial participation might explain that FAT/CD36 expression was also seen in Kupffer cells and smooth muscle cells, which both are affected by radiation and hypoxia.

In fact, fat accumulation is a multifactorial process. Therefore it could be suggested that more than one factor (FAT/CD36) needs to be inhibited to control fat accumulation in the liver. We concentrated on TNF-α, as we know that it is elevated after irradiation and is the main cytokine in our model inducing FAT/CD36 expression, a major fat transporter protein. Further studies will need to be performed to explore the relationship of radiation-induced inflammatory mechanisms and fatty acid uptake with proteins involved in energy metabolism as well as oxidative stress mechanisms following liver irradiation.

## 4. Material and Methods

### 4.1. Materials

All chemicals used were of analytical grade and purchased from commercial sources as follows: real-time polymerase chain reaction (PCR) primers, moloney murine leukemia virus (M-MLV) reverse transcriptase, reverse transcription buffer, 0.1 M dithiothreitol (DTT), and Platinum SYBR green qPCR UDG mix were from Invitrogen (Carlsbad, CA, USA); dNTPs, Protector RNase inhibitor, Klenow enzyme, primer oligo (dT)15 for complementary DNA (cDNA) synthesis, and Salmon sperm DNA were from Roche (Penzberg, Germany); Hybond N nylon membranes were purchased from Amersham Pharmacia Biotech (Amersham, UK), 4,6-diamidino-2-phenylindole (DAPI) from Southern Biotech (Birmingham, AL, USA). All other reagents and chemicals were from Sigma-Aldrich (St. Louis, MO, USA) or Merck (Darmstadt, Germany).

### 4.2. Animal Model

Male mice of C57BL/6J strain of about 20–28 gram body weight were purchased from Charles River Laboratories (Sulzfeld, Germany). Selective mouse liver irradiation was performed percutanously with a single dose of 25 Gy (dose rate of 2.4 Gy/min). A lead protection shield was used to protect the mouse body, exposing only the liver to irradiation. Irradiation was done in the presence/absence of TNF-α/IFX using a RS 225 X-ray Research System from Gulmay Medical Ltd. (Camberley, UK) operating at 200 kV, 15 mA, and with 0.5-mm Copper filtration. Animals were anaesthetized by inhaling Sevoflurane from Abbott GmbH & Co. KG (Wiesbaden, Germany). Sham-irradiated control animals were handled simultaneously. Treated animals and sham-irradiated controls were sacrificed at 1, 3, 6, 12, 24 and 48 h after irradiation. All animals received humane care in accordance to the German Law for the Protection of Animals and the institutional guidelines. The treatment of the mice, and the experiments were approved (approval number: 33.9-42502-04-10/0158 on 20 July 2010) by the local committee of University of Goettingen and public authority on animal welfare.

### 4.3. Animal Groups of Different Treatments

Mice were randomly assigned into 6 groups (see Table 1). Group 1 (G1) were sham-irradiated controls, group 2 (G2) contained mice receiving a single-dose treatment of 25 Gy. Group 3 (G3) and group 4 (G4) were given combined treatment of intraperitoneally administered single injection of TNF-α (2 µg/mouse) or IFX (10 mg/kg), respectively, 20 min before single-dose irradiation of 25 Gy. Group 5 (G5) and group 6 (G6) were given a TNF-α (2 µg/mouse) or IFX dose (10 mg/kg), respectively, however, they did not receive radiation treatment. In preliminary separate experiments with doses of 5 and 10 mg/kg for IFX and 1 and 2 µg/mouse for the TNF-α, the doses were optimized. The best results were obtained with 10 mg/kg for IFX and 2 µg/mouse for TNF-α.

**Table 1:** Different groups of mice based on their treatment used in the study.

Group 1 (G1)	Group 2 (G2)	Group 3 (G3)	Group 4 (G4)	Group 5 (G5)	Group 6 (G6)
Sham-irradiated	25-Gy	25-Gy + TNF-α	25-Gy + IFX	TNF-α	IFX

### 4.4. Staining of Triglycerides in Irradiated Mice Liver Tissue

Triglycerides were stained in unfixed cryostat liver sections (5 µm) from mice by Sudan III from Morphisto (Frankfurt, Germany) to study the histology of irradiated liver tissues at different time points compared to a sham-irradiated control. Sudan III-stained slides were evaluated by using a BX43 light microscope and digital DP21 camera by Olympus (Tokyo, Japan). Counterstaining of the nuclei was performed by Hematoxylin from Morphisto (Frankfurt, Germany).

### 4.5. Immunofluorescent Double-Staining

Immunofluorescence staining was performed as described before [24]. For double-staining monoclonal FAT/CD36 antibody was co-incubated with either polyclonal antibody directed against CK19, polyclonal antibody directed against F4/80 or monoclonal antibody directed against SMA. Cryosections of 5 µm thickness were fixed with acetone/methanol, washed in PBS and subsequently incubated with blocking medium (90% of a 0.1% BSA, 10% FCS in PBS) for 1 h at room temperature. The antibody dilutions were applied over night at 4 °C onto the sections using a FAT/CD36 monoclonal antibody from Abcam (Cambridge, UK) and F4/80 monoclonal antibody from Abcam (Cambridge, UK) CK19 polyclonal antibody from Abcam (Cambridge, UK) and SMA monoclonal antibody from Sigma-Aldrich (St. Louis, MO, USA) respectively. Non-immune serum was used as negative control. (4',6-diamidino-2-phenylindole (DAPI)); SouthernBiotech (Birmingham, AL, USA) served as nucleic acid stain. The slides were observed by using an Axiovert 200M epifluorescence microscope Zeiss (Jena, Germany).

### 4.6. Triglyceride Profile in Serum and Liver Tissue of Mice after Irradiation

Liver tissues and serum samples were collected from mice at the studied time points, tissues were frozen in liquid nitrogen and both were stored at −80 °C. Frozen liver portions (approximately 100 mg) were homogenized in 5% Triton X-100 Merck (Darmstadt, Germany). The concentrations of triglycerides in liver lysate and serum samples were determined by utilizing the automated systems of the central laboratory of the Institute of Clinical Chemistry in University Medical Center Goettingen.

### 4.7. RNA Isolation and Real-Time PCR Analysis

Total RNA from the livers of irradiated and sham-irradiated mice was isolated after homogenization in Trizol^®^ (Invitrogen) according to manufacturers’ protocol. For real-time PCR, reverse transcription of the extracted RNA samples was performed using a Superscript kit from Invitrogen as described previously [24]. Briefly, cDNA was generated by reverse transcription of 1 µg of total RNA using 100 nM of dNTPs, 50 µM of primer oligo dT15, 200 U of moloney murine leukaemia virus reverse transcriptase (M-MLV RT), 16 U of protector RNase inhibitor in RT buffer and 2.5 µL of 0.1 M DTT; real time PCR was performed using a StepOnePlus™ sequence detection system from Applied Biosystems (Darmstadt, Germany) with primer detecting TNF-α-sequence (fwd 5'-CAAACCACCAAGTGGAGGAG-3', rev 3'-GTGGGTGAGGAGCACGTAGT-5'), GAPDH (fwd 5'-AGAACATCATCCCTGCATCC-3', rev 3'-CACATTGGGGGTAGGAACAC-5') and β-actin (fwd 5'-ATTGTTACCAACTGGGACGACATG-3' rev 3'-CGAAGTCTAGAGCAACATAGCACA-5) as housekeeping genes. Fold change expression was calculated using threshold cycle (*C*_t_) values. The primers were synthesized by Invitrogen.

### 4.8. Protein Extraction from Liver Tissue

About 50 mg frozen tissue was homogenized with an Ultra-turrax TP 18/10 from Cole-Parmer (Vernon Hills, IL, USA), three times for 10 s each, in 10 vol 50 mM TRIS-HCl buffer, pH 7.4, containing 150 mM sodium chloride, 1 mM EDTA, 1% Triton X-100, 1 mM phenylmethane sulfonyl-fluoride (PMSF), 1 mM benzamidine, 1 mg/mL leupeptin, 10 mM chymostatin, 1 mg/mL antipain, and 1 mg/mL pepstatin A. The entire procedure was carried out at 4 °C. Crude homogenates were passed five times through a 22G needle attached to a syringe and centrifuged for 5 min at 10,000× *g* at 4 °C. The protein concentration was determined in supernatants by using the BCA (bicinchoninic acid) protein assay reagent kit from Pierce (Rockford, IL, USA). Aliquots of the homogenates were stored at −20 °C until further used for Western blot analysis.

### 4.9. Western Blot Analysis

Samples of 50 μg protein were applied per well and subjected to polyacrylamide gel electrophoresis using NuPAGE 4%–12% Bis-Tris Gel from Invitrogen (Carlsbad, CA, USA) under reducing conditions [40]. After electrophoresis, the proteins were transferred to Hybond-ECL (enhanced chemiluminescence) nitrocellulose membranes [41]. Immunodetection was performed according to the ECL Western blotting protocol described before [28]. The antibody used in this study was monoclonal anti-FAT/CD36 from Abcam (Cambridge, UK). β-actin from Sigma-Aldrich (St. Louis, MO, USA) was used for equal loading.

### 4.10. Statistical Analysis

The data were analyzed using Graph pad Prism 4 software (San Diego, CA, USA). All experimental errors are shown as S.E.M. Statistical significance was calculated by student *t*-test. Significance was accepted at * p < 0.05.

## 5. Conclusions

In summary, selective liver radiation and acute phase processes triggered fat uptake via FAT/CD36 into mice livers from the serum. Accordingly, anti-TNF-α therapy prevented early radiation-induced expression of FAT/CD36 *in vivo*. As TNF-α is an important pro-inflammatory cytokine in irradiation-mediated liver damage and induces FAT/CD36 expression, neutralization of TNF-α using an anti-TNF-α antibody seems to be a strategy to prevent further irradiation damage including metabolic changes.
